# Barriers and facilitators to the implementation of social robots for older adults and people with dementia: a scoping review protocol

**DOI:** 10.1186/s13643-021-01598-5

**Published:** 2021-02-05

**Authors:** Wei Qi Koh, Simone Anna Felding, Elaine Toomey, Dympna Casey

**Affiliations:** 1 School of Nursing and Midwifery, National University of Ireland Galway, Galway, Ireland; 2 DZNE German Center for Neurodegenerative Diseases, Dortmund, Germany; 3 School of Allied Health, University of Limerick, Limerick, Ireland

**Keywords:** Social robots, Implementation, Barriers, Facilitators, Older adults, Dementia

## Abstract

**Background:**

Psychosocial health issues such as depression and social isolation are an important cause of morbidity and premature mortality for older adults and people with dementia. Social robots are promising technological innovations to deliver effective psychosocial interventions to promote psychosocial wellbeing. Studies have reported positive findings regarding this technology on the psychosocial health of older adults and people with dementia. However, despite positive findings of the effects of social robots for older adults and people with dementia, little is known about factors affecting their implementation in practice.

**Methods:**

This study follows Arksey and O’Malley’s approach and methodological enhancement by Levac et al. Relevant articles will be identified by searching electronic databases: MEDLINE, Embase, PsycINFO, Scopus, Web of Science, Compendex and PubMed. A two-phase screening process will be undertaken by two independent reviewers to determine articles’ inclusion. Findings will be summarised and reported thematically based on domains in the Consolidated Framework of Implementation Research (CFIR) and presented narratively. The Preferred Reporting Items for Systematic Reviews and Meta-Analyses extension for scoping reviews (PRISMA-ScR) will guide the reporting of findings.

**Discussion:**

Reporting the protocol in advance of conducting the review will ensure that rigorous and transparent methodological approach is undertaken. The outcomes of the review include identifying variants in terminologies used to describe implementation, identifying the scope of the literature regarding the barriers and facilitators affecting the implementation of social robots and identifying research gaps to guide further empirical research in this field. This evidence synthesis constitutes part of a bigger project aimed to develop implementation guidelines for social robotics for older adults with dementia. Since the methodological process consists of reviewing and collecting data from publicly available data, this study does not require approval from a research ethics board.

**Scoping review registration:**

Our protocol is registered with the Open Science Framework (https://osf.io/2x3y9/) as an open access article, under the Creative Commons Attribution Non Commercial (CC BY-NC-4.0) license, which allows others to distribute, remix, adapt and build on this work on a non-commercial basis, and license their derivative work using different terms, on the basis that the original basis is properly cited and the use is non-commercial (http://creativecommons.org/licenses/by-nc/4.0/).

**Supplementary Information:**

The online version contains supplementary material available at 10.1186/s13643-021-01598-5.

## Background

Psychosocial health issues among older adults, such as depression and social isolation, are an important cause of morbidity and premature mortality for older adults [1, 2]. In particular, older adults with dementia are at a higher risk of developing these issues [3]. Even though people with dementia may want to remain socially connected and be involved in activities that are personally meaningful [4], disease-related impairments such as impaired cognition and emotional control can reduce their capacities and confidence. The stigma surrounding dementia and age discrimination amplifies the isolating effects of disease-related impairments and can have disempowering, dehumanising and marginalising effects on people with dementia [5]. Consequently, they are predisposed to heightened risk of depression and a further decline in cognition and function [6]. As the world’s population is ageing rapidly, these issues are expected to be amplified. It is therefore important to look into effective interventions to promote psychosocial wellbeing of older adults and people with dementia. Technological innovations have been viewed as solutions to deliver effective psychosocial interventions [7], and one such example would be the use of social robots. Social robots are defined as ‘useful robots with social intelligence and skills to allow interaction with people in a socially acceptable manner [8]. According to Góngora and colleagues [9], there are four classifications of social robots: pet robots, humanoid robots, telepresence robots and socially assistive robots.

The effectiveness of social robots on the psychosocial health of older adults has been evaluated in the literature. A recent systematic review of 11 randomised controlled studies, of which 80% of the 1042 participants had dementia or mild cognitive impairment, was conducted by Pu et al. [10]. Social robots were found to improve social engagement between participants and staff, and positively affect physiological indicators of participants through improved sleep, improved oxygenation and improved cardiac status, as well as reduced use of psychotropic drugs for people with severe dementia. Likewise, a scoping review was conducted to map the key benefits of PARO, a robotic seal, for older people with dementia in care settings based on 29 included studies [11]. The findings were congruent to the findings from the systematic review. The first benefit was reduced negative emotions and behavioural symptoms in people with dementia, which includes decreased agitation, less use of psychotropic medications, reduction in wandering behaviour, reduced staff stress and caregiver burn out. Secondly, it helped to improve mood, as caregivers highlighted that users had brighter facial expressions, improved quality of sleep and reduced use of pain medication. Thirdly, it helped to improve visual and verbal social engagement and was used to facilitate conversations between care home residents and staff. The findings from both reviews elucidate evidence on positive trends on the effects of social robots on the psychosocial health of older adults, including people with dementia.

After the effectiveness of interventions have been evaluated, the next phase is to examine their implementation in a real-world setting [12], where conditions for implementing an intervention differ from a research setting. Contexts for research (i.e., clinical) trials are dependent on research-supported resources, have specified timeframe and are transient in nature since interventions are usually discontinued after a trial ends [13]. In contrast, factors that influence implementation in real-world practice, such as competing demands on the care provider, may not be reflected in a research trial [14]. Despite positive findings with regards to the effects of social robots for older adults, little is known about how to ensure that these interventions are implemented in practice. Papadopoulos and colleagues conducted a systematic review of twelve articles to identify enablers and barriers to the implementation of socially assistive humanoid robots (SAHR) in health and social care [15]. The authors found that facilitators include participants’ enjoyment, intuitiveness and ease of use of the SAHR, personalisation of SAHR services to users’ needs, as well as familiarity towards the SAHR. On the other hand, barriers to implementation were limited capabilities of the robot, as well as negative preconceptions about stigma and dehumanisation of care. The authors reported these determinants were identified through single articles due to heterogeneity in study designs; therefore, their findings may not be generalisable. It is also worth noting that the construct of ‘implementation’ was not included in the search strategy. Instead, a broader and less specific construct relating to the ‘context of implementation’ (i.e. health and social settings) was used. This may have limited the specificity of searches, and some pertinent studies might have been missed out. Furthermore, the term ‘implementation’ was not included in the titles or abstracts of any of the included articles. Rather, terms such as ‘service evaluation’, ‘usability’, ‘social acceptance’, ‘acceptability’ and ‘feasibility’ were used. This suggests variations in terminologies used to describe implementation in existing studies.

## Rationale

The recent systematic review by Papadopoulos et al. [15] has provided important insights relating to the implementation of SAHRs. Our review differs in that it encompasses a broader scope to allow inquiry into the implementation of all variants of social robots. Given the variations in terminologies that have been used to describe implementation, this broader scope of evidence synthesis is necessary to establish the breadth of evidence base and to identify knowledge lapses in this field. In addition, our review places a greater focus on the conceptual aspects of implementation. This will be reflected through our search strategy and the use of the Consolidated Framework of Implementation Research (CFIR) to frame our findings. A scoping review is an ideal method for evidence synthesis to meet these goals, since it allows for broad exploration of literature [16, 17]. Moving forward, the findings from this review would facilitate a better understanding of factors that are affecting the implementation of social robots for older adults in real-world practice, and to identify research gaps.

## Objectives

The aim of this review is to facilitate a better understanding of factors that are affecting the implementation of social robots for older adults and people with dementia in real-world practice. The research questions for this review are as follows:

1. What are the terminologies that have been used to describe implementation in relation to social robots?
2. What are the barriers and facilitators affecting the implementation of social robots for older adults, including people with dementia?

## Methods

### Conceptual framework

According to Nilsen, implementation is a multidimensional phenomenon, where implementation barriers and facilitators can be influenced by an interplay of multi-level factors ranging from individual to organisational factors [18]. The Consolidated Framework for Implementation Research (CFIR) is a determinant framework in implementation science that was developed to guide the systematic assessment of multi-level contexts to identify determinants (i.e. factors) that can influence intervention implementation [19]. The constructs in CFIR are derived from the synthesis of theories on dissemination, innovation, organisational change, implementation, knowledge translation and research uptake, and have received consensus from experts in this field [19]. It comprises of 39 constructs grouped within five major domains—intervention, outer setting, inner setting, characteristics of individuals and implementation process—all of which interact to influence intervention implementation and implementation effectiveness. This framework has been previously applied to wide ranging fields of study, including eHealth technology [20], and provides a comprehensive approach to the investigation on barriers and facilitators that can affect implementation. Therefore, results of the extracted articles will be synthesised and integrated using the CFIR. Use of this framework will allow barriers and facilitators affecting implementation of interventions to be identified and presented in a structured manner. It will also enable findings from this review to be more readily comparable to other implementation studies and allow gaps in research to be identified.

## Protocol development

This study follows the Arksey and O’Malley’s [16] framework for scoping reviews along with methodological enhancement by Levac et al. [17]. The five stages within this framework are (1) identifying the research question, (2) identifying relevant studies, (3) selecting studies, (4) charting the data, and (5) collating, summarising and reporting the results. The study protocol was registered with the Open Science Framework on 16 May 2020 (https://osf.io/2x3y9/), under the Creative Commons Attribution Non Commercial (CC BY-NC-4.0) license. This means that others may distribute, remix, adapt and build on this work on a non-commercial basis, and license their derivative work using different terms, on the basis that the original basis is properly cited and the use is non-commercial (http://creativecommons.org/licenses/by-nc/4.0/). The structure and content of this protocol follows the Preferred Reporting Items for Systematic reviews and Meta-analyses extension for Scoping Reviews (PRISMA-ScR) checklist [21] (Additional File 1). The Preferred Reporting Items for Systematic Reviews and Meta-analysis Protocols (PRISMA-P) was not used as many items in the checklist are not applicable to a scoping review.

### Stage 1: identifying the research question

The main research question is defined as: ‘What are the barriers and facilitators that affect the implementation of social robots for older adults, including people with dementia?’ To allow for a broad exploration of research that has been conducted in this field, no limits will be applied to the context of implementation (i.e. study settings).

### Stage 2: identifying relevant studies

#### Search strategy

Relevant published studies or literature will be identified by searching the following electronic databases and search systems: MEDLINE via Ovid, Embase via Ovid, PsycINFO via Ovid, Web of Science Core Collection (via Web of Science), Scopus, Compendex and PubMed. Reference lists of selected studies will be hand searched to ensure that any additional literature that may be of relevance will be identified. To ensure that all relevant information is captured, grey literature sources (e.g. Web of Science Conference Proceedings, Google Scholar) will also be searched to identify studies, reports and conference abstracts of relevance. Several terminologies have been used across the literature to describe the term or concept of implementation [22]. Therefore, to improve the specificity of searches [23], the taxonomy of implementation outcomes that was developed by Proctor and colleagues [24] will be used to guide the systematic search for articles relating to implementation. This taxonomy consists of eight constructs, which includes acceptability, adoption, appropriateness, feasibility, fidelity, implementation cost, penetration and sustainability. Some of these concepts have been used in studies to describe implementation of SAHRs [15]. Therefore, use of these concepts as part of the search strategy will likely yield relevant results and ensure thoroughness of the searches. The concepts in the taxonomy are to guide the literature search strategy, so that the search and selection of articles may be consistent, broad and unbiased. To improve the sensitivity of searches [23], the search strategy will not include terms such as ‘facilitators and barriers’, ‘factors’ or ‘determinants because the authors anticipated that such terms are often not mentioned in the title and/or abstract. Instead, barriers and facilitators to implementation may only be discussed in the body of the text. Hence, this information will only be assessed through reading the full texts at a later phase of screening to ensure that no potentially relevant articles are omitted. This will enable a more thorough overview of all research that implemented social robots for older people and people with dementia. These search strategies were developed in consultation with a research librarian to optimise the specificity and sensitivity of the searches, who will also provide support throughout the search process. No forward citation tracing will be conducted; however, the reference list of relevant reviews and included studies will be hand searched to identify other potentially relevant studies. A sample search strategy that has been developed in consultation with a research librarian can be found in Additional File 2.

### Stage 3: study selection

The titles and abstracts resulting from the search strategy focused on the barriers and/or facilitators that affect the implementation of social robots will be included for review. Articles will be imported into EndNote and be deduplicated. To determine eligibility, a two-phase screening process will be undertaken by two independent reviewers. Firstly, titles and abstracts of identified articles will be screened for eligibility by each reviewer as per the following inclusion criteria: (1) use of social robot(s) as an intervention, (2) involve older adults and/or people with dementia, (3) published in English language, and (4) provide information regarding factors affecting the implementation of social robots, based on any of the constructs listed in the taxonomy of implementation outcomes. This approach of evidence selection will reduce the potential for evidence selection bias [25]. All types of empirical research studies encompassing any types of methods and study designs will be included. No search limits will be applied to the year of publication, and all publications will be searched from inception. Correspondingly, (1) non-empirical studies such as review articles, commentaries or expert opinions, (2) studies that do not involve older adults and/or people with dementia, (3) published in non-English language and (4) do not contain any terms relating to implementation will be excluded. Next, the full texts of relevant papers will be screened. At the end of each screening process, the reviewers will compare their decisions. Any non-consensus or ambiguity regarding eligibility for inclusion will be discussed and resolved among both reviewers and with a third independent reviewer if necessary.

### Stage 4: charting the data

A standardised charting sheet will be developed using Microsoft Excel to allow reviewers to chart the data to confirm the studies’ relevance and to extract their characteristics. Charting refers to the technique of sifting, mapping out and sorting of materials based on their key characteristics [26]. Study characteristics to be extracted will include information such as authors’ name, year of publication, study design, country, participants’ demographics, study setting, construct or term used to describe implementation, and key relevant results relating to the aim of the research question (i.e. barriers and facilitators affecting implementation). This charting sheet will be reviewed and pre-tested by both reviewers to ensure consistency in data extraction and that all the necessary information is captured from each study. Each reviewer will then independently extract data from the included studies, and comparisons will be made afterwards. Any incongruence will be discussed and resolved among both reviewers and with a third independent reviewer if necessary. Finally, the data will be combined into a single Microsoft Excel spreadsheet.

### Stage 5: collating, summarising and reporting the result

In this stage, findings will be collated, summarised and reported. First, terms that were used to describe implementation will be mapped onto Proctor’s taxonomy of implementation outcomes. Terms that are not described in the taxonomy will be identified as independent terms. The frequency in which these terms were used will be presented. Next, the types of social robots used will be categorised into three operational groups based on their functions: (1) socially assistive robots, (2) pet robots and (3) telepresence robots (Additional File 3). Next, to synthesise the extracted data on barriers and facilitators, directed content analysis [27] will be applied deductively using the CFIR [28, 29]. Based on the extracted data, data synthesis will be conducted separately for older adults and people with dementia. Barriers and facilitators will be mapped onto one of the 39 constructs in the CFIR, based on a pre-established codebook of definitions that has been adapted to fit this study (Additional File 4). Although a deductive approach to analysis is planned, open (inductive) coding may be applied to barriers and facilitators that do not fit any of the existing CFIR constructs to generate new constructs and categories. This synthesis will be verified by a second reviewer. Any disagreements will be discussed and resolved among both reviewers and with a third reviewer, as necessary. All data will be organised thematically according to the five domains in the CFIR to map and present implementation barriers and facilitators in a structured manner. This will show areas that have been under researched and may require further investigation. The findings of the study will then be presented narratively [30, 31], using the PRISMA-ScR checklist [21]. Gaps in literature will be discussed, and areas for further research will be identified. A PRISMA flow chart will be used to present the methodological process in detail.

## Discussion

The advance reporting of the scoping review protocol can ensure a rigorous and transparent methodological and conceptual approach [32]. This study will be the first scoping review to conduct a broad exploration of the literature to systematically identify barriers and facilitators affecting the implementation of all variants of social robots for older adults and people with dementia, using a conceptual framework. The findings from this scoping review will help to identify the scope of the literature regarding the barriers and facilitators in relation to the implementation of social robots, for older adults and people with dementia, provide synthesised findings of the results, discuss the implementation terminology used in the literature and identify research gaps to guide further empirical research in this field. This evidence synthesis constitutes part of a bigger project aimed to develop implementation guidelines for using social robots for older adults with dementia.

Even though this review will follow a rigorous method, we anticipate limitations to this review. Firstly, since only publications in English are included, the comprehensiveness of the findings in this review may be limited. Secondly, since this is a scoping review, quality assessment and grading of included studies will not be conducted. Hence, it cannot determine whether the included studies provide robust and generalisable findings.

## Supplementary Information

**Additional file 1.** Preferred Reporting Items for Systematic reviews and Meta-Analyses extension for Scoping Reviews (PRISMA-ScR) Checklist.

**Additional file 2.** Sample search strategy.

**Additional file 3.** Categorisation of social robots.

**Additional file 4.** CFIR Codebook of Definitions.

## Data Availability

Data sharing is not applicable to this article as no datasets were generated or analysed during the current study.

## Abbreviations

SAHR: Socially assistive humanoid robot
CFIR: Consolidated Framework of Implementation Research
