# Supplementary material for: Barriers and facilitators to the implementation of social robots for older adults and people with dementia: a scoping review protocol
Source: Syst Rev. 2021 Feb 5;10:49. doi: 10.1186/s13643-021-01598-5 (PMC7866756; doi:10.1186/s13643-021-01598-5)
Supplement: Supplementary file 2 — Additional file 2. Sample search strategy. [file 13643_2021_1598_MOESM2_ESM.docx]

**PsycINFO Search Strategy**

1. social* AND robot*.mp.

2. Aged/

3. (age or elderly or senior citizen* or older adult).mp. [mp=title, abstract, original title, name of substance word, subject heading word, floating sub-heading word, keyword heading word, organism supplementary concept word, protocol supplementary concept word, rare disease supplementary concept word, unique identifier, synonyms]

4. dementia.mp. or Dementia/

5. implement*.mp.

6. quality improvement.mp. or Quality Improvement/

7. dissemination.mp. or Information Dissemination/

8. "Patient Acceptance of Health Care"/ or acceptability.mp.

9. satisfaction.mp. or Personal Satisfaction/

10. adoption.mp.

11. uptake.mp.

12. "Delivery of Health Care"/ or utili*ation.mp.

13. appropriateness.mp.

14. cost.mp. or "Costs and Cost Analysis"/

15. Feasibility Studies/ or feasib*.mp.

16. fidelity.mp.

17. sustainability.mp. or Program Evaluation/

18. penetration.mp.

19. 2 or 3 or 4

20. 5 or 6 or 7 or 8 or 9 or 10 or 11 or 12 or 13 or 14 or 15 or 16 or 17 or 18

21. 1 and 19 and 20
