# Supplementary material for: Barriers and facilitators to the implementation of social robots for older adults and people with dementia: a scoping review protocol
Source: Syst Rev. 2021 Feb 5;10:49. doi: 10.1186/s13643-021-01598-5 (PMC7866756; doi:10.1186/s13643-021-01598-5)
Supplement: Supplementary file 3 — Additional file 3. Categorisation of social robots. [file 13643_2021_1598_MOESM3_ESM.docx]

**Additional File 3 - Categorisation of social robots**

There have been several proposed ways of categorising social robots based on different attributes (1). For example, Fong et al (2) suggested four ways in which social robots could be classified based on their morphology: anthropomorphic (human-like), zoomorphic (animal-like), caricatured (cartoonish) and functional. Gongora et al (3) proposed the categorisation of social robots into four different categories: (i) pet robot, (ii) humanoid robot, (iii) social assistive robot, (iv) telepresence robot (5). The authors provided examples of social robots in each category; however the definitions and explanation of each category was not elaborated in detail. It is also unclear if the social robots were classified based solely on their morphology or function. For example, the categories ‘pet robot’ and ‘humanoid robot’ appears to be categorising them based on their appearances, while ‘social assistive robot’ appears to be categorised based on their function. In addition, categories – such as “humanoid robot” and “socially assistive robot” - do not appear to be be mutually exclusive. Hence, some subjectivities may be introduced if using this framework for classifying social robots.

The intention of the following categorisation of social robots (Table 1) is for the practical purpose of a categorising social robots for a scoping review (4). Social robots will be categorised into three operational groups based on their functions:

**Table 1: Categorization of social robots based on their functions**

|  | **Type of social robots** | **Functions** |
| --- | --- | --- |
| 1 | Socially assistive robots | Social robots with functions to assist users with tasks (5) |
| 2 | Pet robots | Viable substitutes to live animals (6) and functions as pet therapy to provide physiological and emotional benefits for users (7) |
| 3 | Telepresence robots | Has a video conferencing system mounted on a mobile robotic base, and has a primary function to provide social interaction between humans (8). |

**References**

1. Čaić M, Mahr D, Oderkerken-Schröder G. Value of social robots in services: social cognition perspective. Journal of Services Marketing. 2019.

2. Fong T, Nourbakhsh I, Dautenhahn K. A survey of socially interactive robots. Robotics and autonomous systems. 2003;42(3-4):143-66.

3. Góngora Alonso S, Hamrioui S, de la Torre Díez I, Motta Cruz E, López-Coronado M, Franco M. Social robots for people with aging and dementia: a systematic review of literature. Telemedicine and e-Health. 2019;25(7):533-40.

4. Koh W, Felding S, Toomey E, Casey D. Barriers and Facilitators to the Implementation of Social Robots for Older Adults and People with Dementia: A Scoping Review Protocol. 2020.

5. Feil-Seifer D, Mataric MJ, editors. Defining socially assistive robotics. 9th International Conference on Rehabilitation Robotics, 2005 ICORR 2005; 2005: IEEE.

6. Leng M, Liu P, Zhang P, Hu M, Zhou H, Li G, et al. Pet robot intervention for people with dementia: a systematic review and meta-analysis of randomized controlled trials. Psychiatry Research. 2019;271:516-25.

7. Abbott R, Orr N, McGill P, Whear R, Bethel A, Garside R, et al. How do “robopets” impact the health and well‐being of residents in care homes? A systematic review of qualitative and quantitative evidence. International Journal of Older People Nursing. 2019;14(3):e12239.

8. Stahl C, Anastasiou D, Latour T, editors. Social Telepresence Robots: The role of gesture for collaboration over a distance. Proceedings of the 11th PErvasive Technologies Related to Assistive Environments Conference; 2018.
